# Membrane and synaptic defects leading to neurodegeneration in *Adar* mutant *Drosophila* are rescued by increased autophagy

**DOI:** 10.1186/s12915-020-0747-0

**Published:** 2020-02-14

**Authors:** Anzer Khan, Simona Paro, Leeanne McGurk, Nagraj Sambrani, Marion C. Hogg, James Brindle, Giuseppa Pennetta, Liam P. Keegan, Mary A. O’Connell

**Affiliations:** 1 CEITEC Masaryk University, Kamenice 735/5, A35, CZ 62 500 Brno, Czech Republic; 2 National Centre for Biomolecular Research, Faculty of Science, Masaryk University, Kamenice 5, 625 00 Brno, Czech Republic; 3 MRC Human Genetics Unit, Institute of Genetics and Molecular Medicine at the University of Edinburgh, Crewe Road, Edinburgh, EH4 2XU UK; 4 Centre for Integrative Physiology, Euan MacDonald Centre for Motor Neurone Disease Research, Hugh Robson Building, University of Edinburgh, George Square, Edinburgh, EH8 9XD UK

**Keywords:** RNA editing, ADAR, *Drosophila*, Neurodegeneration, TOR, Autophagy

## Abstract

**Background:**

In fly brains, the *Drosophila* Adar (adenosine deaminase acting on RNA) enzyme edits hundreds of transcripts to generate edited isoforms of encoded proteins. Nearly all editing events are absent or less efficient in larvae but increase at metamorphosis; the larger number and higher levels of editing suggest editing is most required when the brain is most complex. This idea is consistent with the fact that *Adar* mutations affect the adult brain most dramatically. However, it is unknown whether *Drosophila* Adar RNA editing events mediate some coherent physiological effect. To address this question, we performed a genetic screen for suppressors of *Adar* mutant defects. *Adar*^*5G1*^ null mutant flies are partially viable, severely locomotion defective, aberrantly accumulate axonal neurotransmitter pre-synaptic vesicles and associated proteins, and develop an age-dependent vacuolar brain neurodegeneration.

**Results:**

A genetic screen revealed suppression of all *Adar*^*5G1*^ mutant phenotypes tested by reduced dosage of the *Tor* gene, which encodes a pro-growth kinase that increases translation and reduces autophagy in well-fed conditions. Suppression of *Adar*^*5G1*^ phenotypes by reduced Tor is due to increased autophagy; overexpression of *Atg5*, which increases canonical autophagy initiation, reduces aberrant accumulation of synaptic vesicle proteins and suppresses all *Adar* mutant phenotypes tested. Endosomal microautophagy (eMI) is another Tor-inhibited autophagy pathway involved in synaptic homeostasis in *Drosophila*. Increased expression of the key eMI protein Hsc70-4 also reduces aberrant accumulation of synaptic vesicle proteins and suppresses all *Adar*^*5G1*^ mutant phenotypes tested.

**Conclusions:**

These findings link *Drosophila Adar* mutant synaptic and neurotransmission defects to more general cellular defects in autophagy; presumably, edited isoforms of CNS proteins are required for optimum synaptic response capabilities in the brain during the behaviorally complex adult life stage.

## Background

*Drosophila melanogaster* has a single *Adar* (adenosine deaminase acting on RNA) gene encoding an orthologue of the vertebrate ADAR2 RNA editing enzyme [1]. In both vertebrates and *Drosophila*, ADAR RNA editing in CNS transcripts is targeted to pre-mRNA exons that form RNA duplexes with flanking intron sequences. Editing events are frequently located in coding regions, leading to the generation of alternative edited and unedited isoforms of CNS proteins (for review [2]). ADAR2 in mammals is required for editing a glutamine codon to arginine at the *Gria2 Q/R* site in the transcript encoding a key glutamate receptor subunit [3]. This editing event regulates the calcium permeability of AMPA class glutamate receptors, and loss of this editing event leads to seizures and neuronal cell death. Thus, mice lacking *Adar2* die within 3 weeks of birth; however, *Adar2; Gria2*^*R*^ transgenic mice with the chromosomal *Gria2* gene mutated to encode arginine are normal indicating that *Gria2 Q/R* is the key editing site in vertebrates [4]. The number of edited transcripts and edited sites is very much greater in *Drosophila* than in vertebrates. Editing site recognition is conserved; human ADAR2 expressed in *Drosophila* rescues *Adar*^*5G1*^ null mutant phenotypes [5] and correctly edits hundreds of *Drosophila* transcripts encoding ion channels subunits and other CNS proteins [6–10].

Our hypothesis is that during the evolutionary increase in site-specific RNA editing events in advanced insects, there has been selection for editing events that allow production of alternative edited and unedited isoforms of CNS proteins [11]; edited isoforms are also more abundant in adult brains than in larval brains in *Drosophila*. RNA editing has also been evolutionarily expanded in cephalopod molluscs [12], consistent with the idea that more RNA editing may be able to enhance some brain function(s). Recent results reveal the complexity of RNA editing in *Drosophila* neurons, showing that different neuronal populations have distinct editing signatures [13]. The extreme opposite hypothesis to ours, that editing events are evolutionary accidents, appears less likely since many editing events are well conserved within insects or cephalopods, respectively, and are under positive selection during evolution [14]. However, it is still possible that the many different editing events serve many different and unconnected purposes. We set out to define the key effects of *Drosophila* Adar RNA editing by identifying genetic suppressors of *Adar* null mutant phenotypes and determining the mechanisms of action of these suppressors.

*Adar* expression increases strongly at pupation, and the number of edited sites and editing efficiencies at most sites is higher after metamorphosis in the brain of the adult fly [6, 15]. In *Drosophila*, transcripts with high and conserved editing include *paralytic* (*para*) [16], *shaker*, *shaker b*, and *cacophony* (*cac*) [17] transcripts which encode the pore-forming subunits of axonal voltage-gated sodium, potassium, or calcium channels, respectively. At the axon terminus, presynaptic active zones are formed above *cacophony* channels clustered in the presynaptic membrane; in the active zones, neurotransmitter synaptic vesicles are tethered for rapid neurotransmitter release followed by rapid endocytosis to recycle and refill the vesicles [18]. The *cacophony* channel triggers calcium entry into presynaptic boutons when it is activated in response to an action potential [19]. Other transcripts that are edited, especially in the adult brain, such as *Synapsin* [20], *Synaptotagmin 1*, *Endophilin A*, and *Munc* [21], encode key proteins involved in the formation and function of neurotransmitter synaptic vesicles.

The *Drosophila Adar*^*5G1*^ null mutant fly shows reduced viability, lack of locomotion, ataxia, and age-related neurodegeneration [6]. In larval motor neurons, targeted *Adar* RNAi knockdown leads to increased motor neuron excitability; reciprocally, *Adar* overexpression in motor neurons leads to reduced neuronal excitability [22]. *Adar*^*5G1*^ mutant larval neuromuscular junctions have defects in calcium-regulated synaptic transmission and increased numbers of boutons [23] with increased numbers of synaptic vesicles and increased levels of the pre-synaptic proteins Synapsin [20], Endophilin A, Synaptotagmin 1, and others [24]. A much weaker hypomorphic *Adar*^*hyp*^ mutant that has a nearly normal capacity for locomotion when stimulated exhibits an aberrantly increased sleep pressure associated with the inability to achieve a normal sleep-mediated reduction of pre-synaptic vesicles and associated proteins and synaptic signaling [25]. This defective locomotion due to persistent halting in the hypomorphic *Drosophila Adar*^*hyp*^ mutant is similar to what we observe in the more severely affected *Adar*^*5G1*^ null mutant. In the *Adar*^*hyp*^ adult brain, the sleep defect is due to neuronal excesses of neurotransmitter synaptic vesicles held in a reserve pool that is not readily releasable and difficult to deplete, and the level of presynaptic proteins is elevated, consistent with defects in axonal active zones in brain neurons similar to those observed at larval neuromuscular junctions [25].

To elucidate whether *Adar* null mutant phenotypes have a coherent underlying basis, we performed a pilot genetic screen on chromosome II for suppressors of the *Adar*^*5G1*^ null mutant reduced viability. We find that reduced dosage of *Tor* (*target of rapamycin*) is a potent suppressor of *Adar* mutant phenotypes. Tor is a member of the phosphatidylinositol 3-kinase-related kinase family and is essential for several cellular processes including increased translation and reduced autophagy under well-fed conditions (for review [26, 27]). Electron microscopic analysis reveals that neurodegeneration in *Adar*^*5G1*^ mutant fly retina is associated with abnormal, large, intracellular membrane-bounded vacuoles. These vacuoles appear to contain cellular components and are likely to result from aberrant activity of the endosome/autophagy/lysosome system. Tor protein levels are increased in the *Adar*^*5G1*^ mutant, and reducing *Tor* gene dosage suppresses these defects by increasing autophagy and clearing excess pre-synaptic proteins. There is no extensive cell death in the *Adar*-mutant CNS. The findings are consistent with the hypothesis that *Drosophila Adar* function has an evolutionarily selected biological role related to synaptic plasticity and CNS protection.

## Results

### Reduced *Tor* gene dosage suppresses *Adar* mutant reduced viability, open field locomotion defects, and reduced longevity

To elucidate which mechanisms mediate *Adar* mutant phenotypes, we performed a pilot screen for heterozygous deletions that increase the number of adult male *Adar*^*5G1*^ flies eclosing from pupae in crosses (*Adar* is on Chr. X and males have one gene copy). When virgin female *y,Adar*^*5G1*^*,w /FM7, Bar* flies are crossed with male *w*^*1118*^ and male progeny that eclose from pupae are counted, the ratio of male *y,Adar*^*5G1*^*,w* to male *FM7 Bar* progeny obtained is only about 20% (see *w*^*1118*^ control cross at the bottom of Additional file 1: Figure S1). This reduced viability at eclosion from the pupa reflects the death of *Adar*^*5G1*^ mutants during embryonic, larval, and pupal stages. Therefore, when virgin female *y,Adar*^*5G1*^*,w /FM7, Bar* flies are crossed with male *w, Df(2)/SM5 Cy*, suppression of this *Adar*^*5G1*^ reduced viability, measured by the proportion of live *Adar*^*5G1*^*; Df(2)/+* mutant flies eclosing from pupae can be used for a genome-wide screen of deficiencies.

We performed a trial screen of 35 *DrosDel* deficiencies [28] covering 70% of the left arm of chromosome II for deficiencies that when heterozygous act as suppressors of the reduced viability of male *Adar*^*5G1*^mutant flies (Additional file 1: Figure S1). *DrosDel* deficiencies are a series of genetically engineered deficiencies covering most of the *Drosophila* euchromatin that each deletes about 30 genes on average [28]. The most robustly rescuing deficiency identified by the screen, *Df(2 L)ED778*, substantially increases (to 80%), and the partially overlapping *Df(2 L)ED784* deficiency somewhat increases, *Adar*^*5G1*^ mutant viability. The viability of *Adar*^*5G1*^ is increased by 8 deficiencies and decreased by others. The level of suppression differs greatly between deficiencies, with many giving slight suppression that makes the results noisy and not ideal for a larger genome-wide screen. As we obtained a robust result from two deficiencies in this pilot screen, we decided to study these further.

**Fig. 1:**
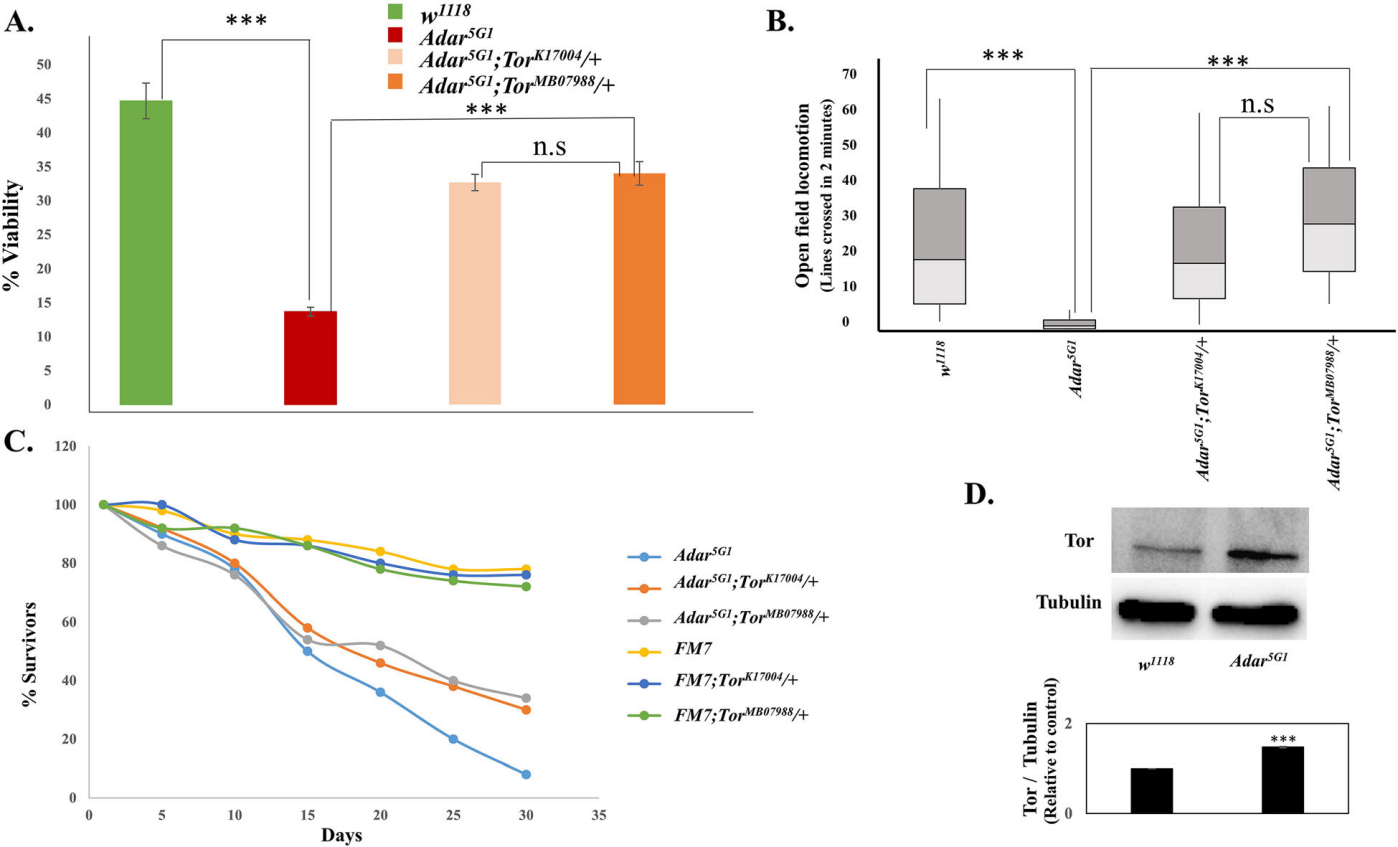
Reduced *Tor* gene dosage rescues *Adar*^*5G1*^ mutant phenotypes. *Tor* mutations increase **a** viability at eclosion from the pupae, *n* = 3; **b** open field locomotion, *n* > 8; and **c** lifespan in *Adar*^*5G1*^mutant flies. *FM7* is a first chromosome balancer strain. *n* = 3. **d** Immunoblot with antibody to *Drosophila* Tor protein of *Adar*^*5G1*^mutant and wild-type (*w*^*1118*^) fly head protein extracts. *n* = 3. Quantitation of immunoblot data shows increased level of Tor in *Adar*^*5G1*^. *p* values in **a** and **b** were calculated by a one-way ANOVA followed by Tukey’s test. The significance of differences between variables was described based on *p* values: *p value < 0.05, **p value < 0.005, ***p value < 0.001, and n.s (not significant). Error bars: SEM (standard error of mean for biological replicates). *p* values in **d** were calculated by Student’s *t* test. Source data values are included in Additional file 6

We tested mutations in individual genes within the rescuing *Df(2 L)ED778* deficiency and the partially overlapping *Df(2 L)ED784* deficiency, and within some other partially rescuing deficiencies, for rescue of *Adar* mutant viability. *DrosDel* deletions are excellent for rapid genome coverage in genetic screens, but for unknown reasons, inability to map effects of deletions down to reduced copy numbers of single genes within the deletions is very common. In this case, single gene mutations in the *Tor* gene, but not mutations in other genes within the deleted regions, were found to increase viability (Fig. 1a) and open field locomotion (Fig. 1b) [29, 30] in *Adar*^*5G1*^*;Tor*^*k17004*^ / + and *Adar*^*5G1*^*;Tor*^*MB07988*^ / + flies; lifespan also appears to be increased (Fig. 1c) (we are unable to perform the appropriate Kolmogorov-Smirnov test for statistical significance with our small sample size in 3 replicates). These *Tor* mutants are homozygous lethal P-element insertions at different positions in *Tor* that are presumed null mutants.

Open field locomotion was measured by recording crossing of individual flies over lines in a gridded Petri dish (three 2-min measurements on each of 10 or more flies for each line) as previously described [17]. In this assay, even wild-type flies may stop moving for part of the 2-min measurement period. However, the *Adar* mutant flies tend to stop within a few tens of seconds and to not move again thereafter. The *Adar*^*5G1*^ mutant flies also show leg tremors and difficulty in walking straight without stumbling (Additional file 7: Video S1 show *Adar*^*5G1*^ mutant walking defects, and Additional file 8: Video S2 shows rescue in *Adar*^*5G1*^*; Tor*^*MB07988*^ / +).


**Additional file 7.** Video of *Adar*^*5G1*^ null mutant showing locomotion defect.



**Additional file 8**. Video of Adar^*5G1*^; *Tor*^*MB07988*^ Double mutant, which shows locomotion defect is recued when *Tor* dosage is reduced in the *Adar* null mutant background.


Reduced *Tor* gene dosage may directly correct an aberrantly increased activity of Tor in *Adar*^*5G1*^. Immunoblot analysis of *Adar*^*5G1*^ mutant total head protein extracts show that Tor protein is present at a significantly increased level in *Adar*^*5G1*^ (Fig. 1d). Increased Tor protein is likely to lead to increased levels of activated Tor but, unfortunately, there is no available antibody to detect specifically the active, phosphorylated form of *Drosophila* Tor.

### Reduced *Tor* gene dosage also suppresses *Adar* mutant age-dependent neurodegeneration

**Fig. 2:**
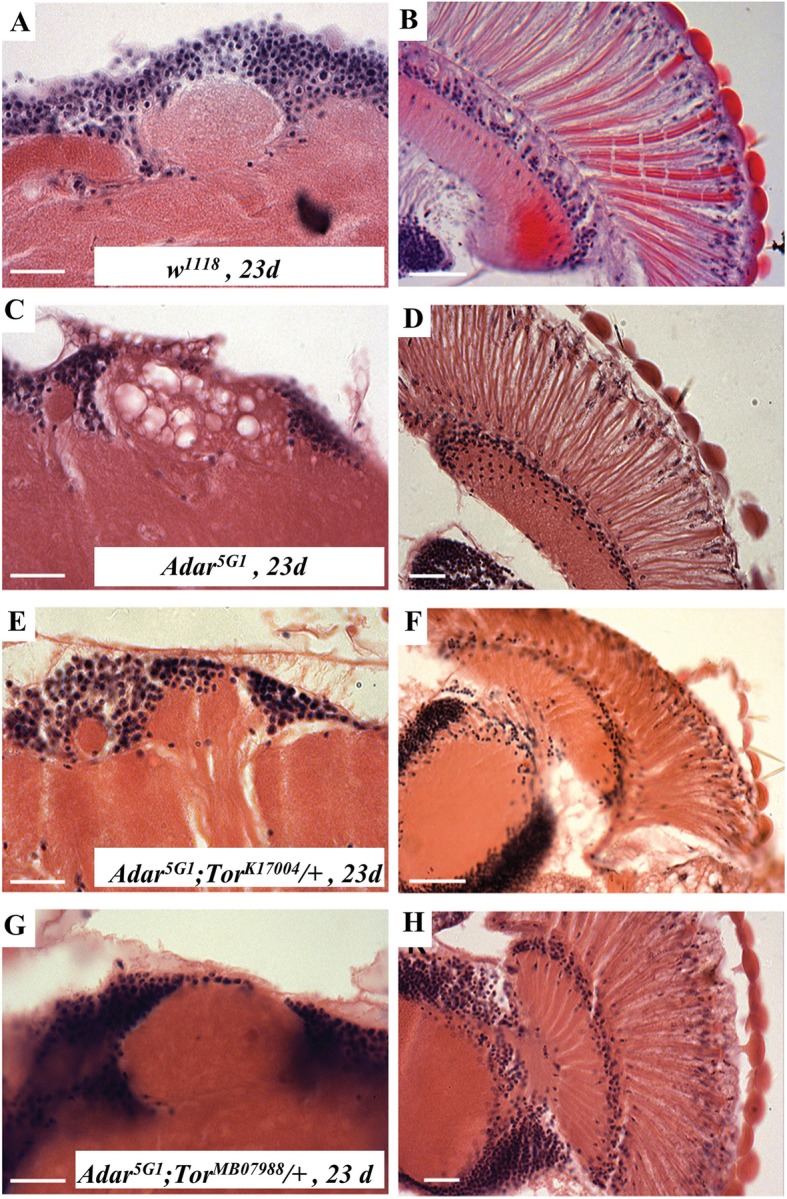
Rescue of *Adar*^*5G1*^ mutant neurodegeneration by reduced *Tor* gene dosage. Images show representative 6-μm-thick hematoxylin and eosin stained sections through mushroom body calyces (left panels (× 63)) and retinas (right panels (× 40)) of **a**, **b** 23-day *w*^*1118*^, **c**, **d** 23-day *Adar*^*5G1*^**e**, **f** 25-day *Adar*^*5G1*^; *Tor*^*K170048*^*/ +*, and **g**, **h** 23-day *Adar*^*5G1*^; *Tor*^*MB07988*^*/ +*. Scale bars, 20 μm

The *Adar*^*5G1*^ null mutant neurodegeneration has been described previously [5, 6, 8, 31]. The *Drosophila* ADAR protein is normally present in nuclei of all brain neurons in wild type and is entirely absent in the *Adar*^*5G1*^ null mutant that deletes the entire *Adar* transcribed region [6]. Neurodegeneration develops more quickly in certain brain regions. In brains of 23-day and 30-day *Adar*^*5G1*^ mutant flies, the calyces of the mushroom bodies (MB) and the retina (Fig. 2c, d, Additional file 2: Figure S2) show filled vacuoles not observed in 23-day *w*^*1118*^ flies (Fig. 2a, b). Within the retina, neurodegeneration is evident at 23 days as a narrowing of photoreceptors with separations appearing between ommatidia (Fig. 2d, Additional file 2: Figure S2). Heterozygous *Tor* mutations suppress the *Adar* mutant neurodegeneration in retina and mushroom body neuropil in *Adar*^*5G1*^*;Tor*^*k17004*^ / + (Fig. 2e, f) and *Adar*^*5G1*^*;Tor*^*MB07988*^ / + (Fig. 2g, h). Neurodegeneration in the *Adar*^*5G1*^ null mutant is 100% penetrant and is never observed in the brain of wild-type flies. We do not quantitate the number of the vacuoles as their size variation is too large; instead, we state whether it occurs or not.

Prominent vacuoles in the brain appear particularly in the mushroom body (MB) calyces. The mushroom body calyces are neuropil regions composed of olfactory projection neuron axons and dendrites of mushroom body Kenyon cells; the cell bodies of the Kenyon cells are located above the calyces and their nuclei stain darkly with hematoxylin. Vacuoles may develop within the large boutons at the pre-synaptic boutons of olfactory projection neurons which extend axons from the olfactory lobes beneath the brain reach to the mushroom body calyces [32]. Large round boutons at the ends of projection neuron axons are surrounded by many fine Kenyon cell dendrites. Both olfactory projection neurons and Kenyon cells have now been shown to be cholinergic [33], consistent with our earlier observations that *Adar*^*5G1*^*; ChAT>Adar 3/4* flies expressing active ADAR under *choline acetyltransferase ChAT*-*GAL4* driver control in cholinergic neurons [34] show rescue of vacuolization in MB calyces and retinas of 30-day *Adar*^*5G1*^ brains [1, 17, 35].

### The *Adar* mutant neurodegeneration involves aberrant membrane processes and formation of large brain vacuoles

**Fig. 3:**
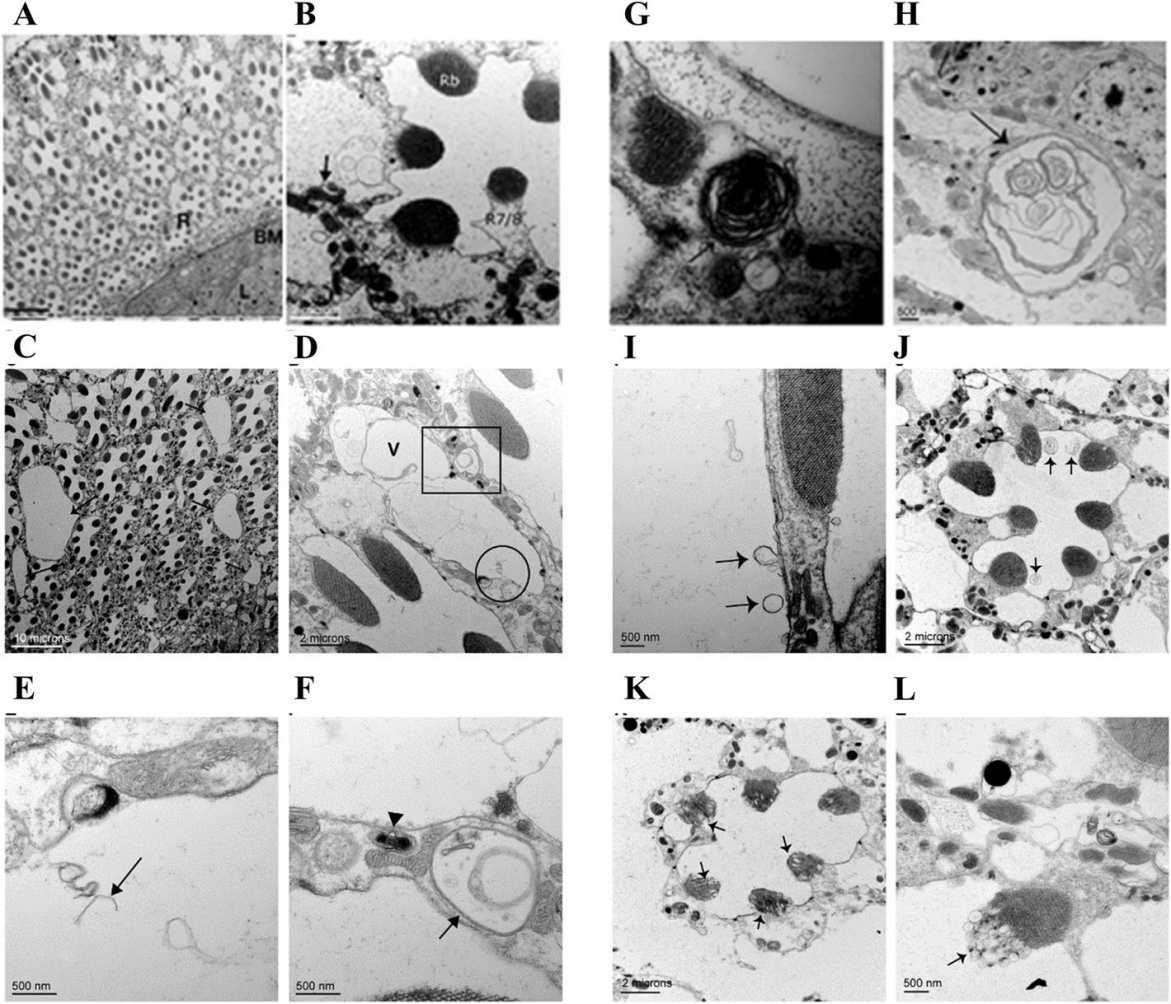
EM analysis of retinal degeneration in the *Adar*^*5G1*^ mutant. **a** The ommatidia of *w*^*1118*^ at 25 days. Each ommatidium comprises seven photoreceptor cells surrounded by and separated from neighboring ommatidia by thin pigment cells containing red pigment granules. **b** An ommatidium of 25-day-old *w*^*1118*^ at higher resolution. The photoreceptor cells with light-detecting rhabdomeres (Rb) appear normal. The R7/R8 photoreceptor is indicated. Organelles such as mitochondria are identifiable (arrow). **c** Retina of the *Adar*^*5G1*^ mutant at 25 days showing pigment cells with large vacuoles between ommatidia (arrows). **d** Higher resolution image of a single ommatidium in 25-day-old *Adar*^*5G1*^ with vacuole (V) between photoreceptor cells of two ommatidia. **e** Magnification of area within the circle in **d**. Interrupted membrane (arrow) was observed inside the vacuole. **f** Magnification of area within the square in **d**. Membrane-bounded vesicles (arrows) in the photoreceptors contain cellular components in an autophagosome-like structure surrounded by two or more membrane layers. **g**, **h** Multilamellar membrane structures (arrows) in a photoreceptor cell and within a glial cell close to the basement membrane between the retina and the lamina in *Adar*^*5G1*^. **i** Single membrane-bounded vesicles pinching off from the photoreceptor (arrows) in early stages of photoreceptor degeneration in *Adar*^*5G1*^. **j** Larger multilamellar membrane structures budding off from the extracellular membrane of photoreceptor cells into the ommatidial cavity (arrows) at more advanced stages of degeneration in *Adar*^*5G1*^. **k** Extensive loss of pigment cells separating ommatidia in advanced stages of neurodegeneration in *Adar*^*5G1*^. Photoreceptor cell cytoplasm and extracellular membrane are abnormal, and vesicles bud from the rhabdomeres (arrows). **l** Abnormal exocytosis from the rhabdomere in late stages. The extracellular membrane of the photoreceptor is not well defined

What is the defect underlying the *Adar*^*5G1*^ mutant neurodegeneration that is strongly suppressed by reduced *Tor* dosage? To examine the *Adar*^*5G1*^ mutant neurodegeneration at higher resolution, we performed an electron microscopic analysis of retinas and optic laminae of aged *Adar*^*5G1*^ mutant flies. Transmission electron microscope (TEM) sections parallel to the surface of the eye are particularly suitable for study because these sections show a highly regular pattern of photoreceptors and support cells within the repeating ommatidia (Fig. 3a, b). TEM images of sections through the retina of 25-day-old *Adar*^*5G1*^ show large membrane-bounded vacuoles between or within support cells that surround the photoreceptors (R1-R7/8) (Fig. 3c, arrows). Other defects in *Adar*^*5G1*^ resemble those seen with autophagy mutants, such as autophagic-like vesicles (Fig. 3d–f), multilamellar vesicles (Fig. 3g, h), and membrane-bounded vesicles budding from the rhabdomeres of photoreceptors in more advanced stages of degeneration (Fig. 3i–l).

This data suggests that the *Adar* mutant neurodegeneration does not involve death of neurons in the first instance, but it does reflect development and enlargement of aberrant intracellular vacuoles like those observed in lysosomal storages diseases that cause defects in autophagy. It is likely that the aberrant vacuoles between ommatidia develop within the retinal pigment cells that import red and brown pigment precursors from the hemolymph and process and store them in membrane-bounded pigment granules that are a type of lysosome-related organelle. We did not obtain TEM sections through mushroom body calyces, but sections through the optic lamina where the cellular arrangements are more difficult to interpret in EM also show aberrant multilamellar vesicles and membrane overgrowth.

Aberrant intracellular membrane processes typify the *Adar* mutant neurodegeneration, which does not appear to involve extensive neuronal death. TUNEL assays did not detect neuronal death in the *Adar*^*5G1*^ mutant brain (Additional file 3: Figure S3A-D), and few Lysotracker-positive nuclei are seen in the brain (Additional file 3: Figure S3B), although cell death does occur outside the brain in head fat cells (Additional file 3: Figure S3A-D). *Adar*^*5G1*^*;ChAT>p35* flies expressing the viral anti-apoptotic protein p35, which inhibits most *Drosophila* caspases [36, 37], still show vacuolization in the MB calyces and retina at 30 days (Additional file 2: Figure S2E, F), indicating that vacuolization is not prevented by blocking apoptosis.

### Suppression of *Adar* mutant phenotypes by reduced *Tor* or by increased expression of Atg5

We next focused on understanding the mechanism of suppression of *Adar* mutant phenotypes by reduced *Tor* gene dosage. *Tor* is a key gene controlling growth and autophagy [27]; suppression of *Adar* mutant phenotypes by reduced *Tor* gene dosage may be due to decreased translation or to increased autophagy in the *Adar*^*5G1*^*; Tor* / + flies.

**Fig. 4:**
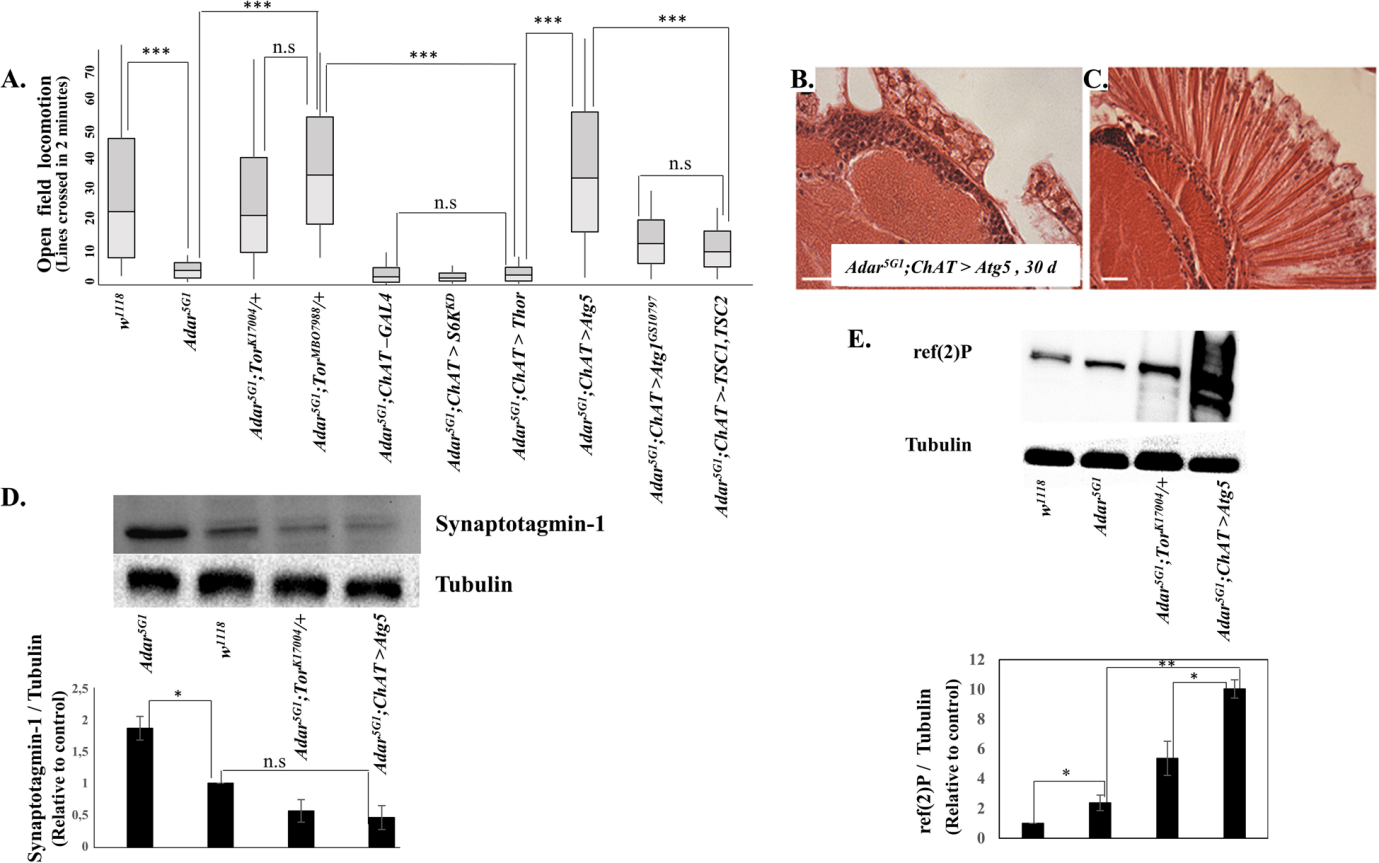
Decreased Tor, or increased Atg5 to increase autophagy, suppresses *Adar*^*5G1*^ mutant phenotypes. **a** Rescue of *Adar*^*5G1*^ mutant open field locomotion defects in *Adar*^*5G1*^; *Tor*^*K170048*^*/ +*, *Adar*^*5G1*^; *Tor*^*MB07988*^*/ +*, *Adar*^*5G1*^*; ChAT>Atg5*, and *Adar*^*5G1*^*; ChAT>Atg1* flies but not in *Adar*^*5G1*^*; ChAT>Thor* or *Adar*^*5G1*^*; ChAT>S6K*^*KD*^ and very partially in *Adar*^*5G1*^*; ChAT>TSC1,TSC2* flies. *n* > 8. **b** Representative images of MB calyx (× 63) and **c** retina (× 40) in 30-day *Adar*^*5G1*^; *ChAT>Atg5*. Scale bars, 20 μm. **d** Immunoblot with antibody to Synaptotagmin 1 of head protein extracts of *Adar*^*5G1*^, *w*^*1118*^, *Adar*^*5G1*^; Tor^*K17004*^ / +, and *Adar*^*5G1*^*; ChAT > Atg5* flies. Quantitation of immunoblot data shows increased Synaptotagmin 1 in *Adar*^*5G1*^ is reduced by decreased *Tor* or by increased *Atg5*. *n* ≤ 3. **e** Immunoblot with antibody to ref(2)p, the *Drosophila* p62 canonical autophagy protein, of head protein extracts of *w*^*1118*^, *Adar*^*5G1*^mutant, *Adar*^*5G1*^; Tor^*K17004*^ / +, and *Adar*^*5G1*^*; ChAT > Atg5* flies. Quantitation of immunoblot data shows that increased ref(2)p, *Drosophila* p62 protein, in *Adar*^*5G1*^ is not reduced but increased by decreasing *Tor* or by increasing *Atg5*. *n* ≥ 3. *p* values were calculated by a one-way ANOVA followed by Tukey’s test. The significance of differences between variables was described based on *p* values: *p value < 0.05, **p value < 0.005, ***p value < 0.001, and n.s (not significant). Error bars: SEM (standard error of mean for biological replicates). Source data values are included in Additional file 6

Tor is a protein kinase that, when active, increases translation by phosphorylation of the ribosomal protein S6 kinase (S6K) protein that increases its activity and by phosphorylation of the eIF 4E BP translation inhibitor that reduces its inhibitory activity [38, 39]. Reduced *Tor* gene dosage should reduce translation in the *Adar*^*5G1*^*; Tor/+* double mutants. However, mimicking translation-decreasing effects of reduced *Tor* gene dosage by decreasing S6 kinase activity in cholinergic neurons in *Adar*^*5G1*^*; ChAT>S6K*^*KQ*^ flies expressing a dominant negative S6K [40], or *Adar*^*5G1*^*; ChAT>Thor* flies with increased expression of translation-inhibiting eIF 4E-BP (*Thor*), did not show suppression of *Adar*^*5G1*^ mutant open field locomotion (Fig. 4a). This indicates that reduced translation is not the primary mechanism by which reduced *Tor* suppresses the *Adar* mutant phenotypes.

Since suppression of the *Adar* mutant phenotypes by reduced *Tor* does not appear to be due to reduced translation, the suppression may instead be due to increases in some type of autophagy. Increased autophagy could be consistent with the clearing of the large vacuoles in aged *Adar* mutant brains and retinas by reduced *Tor* dosage. Activated Tor suppresses autophagy by phosphorylating Atg1, the key protein for activation of canonical autophagy. Increased expression of key autophagy proteins is able to increase canonical autophagy [27]; *Adar*^*5G1*^*; ChAT>Atg5* flies [41] show increased viability and rescue of *Adar*^*5G1*^ mutant locomotion defects (Fig. 4a) and neurodegeneration (Fig. 4b, c). Therefore, suppression of *Adar*^*5G1*^ mutant phenotypes appears to be due to increased autophagy caused by the reduced *Tor* gene dosage.

Tor is activated by growth-promoting extracellular signals such as insulin as well as by intracellular signals; Tor locates to the surface of the lysosome and is activated there by amino acids being returned from the lysosome to the cytoplasm. The insulin receptor acts through PI3 kinase (PI3K) and the serine-threonine protein kinase AKT to phosphorylate the Tuberous Sclerosis Complex (TSC), releasing it from the Rheb (Ras homolog enriched in brain) protein in the lysosomal Tor protein complex and activating Tor [42]. If reduced *Tor* gene dosage suppresses *Adar* mutant phenotypes because it reduces effects of growth-promoting signals such as insulin, then the effect of reduced *Tor* gene dosage should be mimicked by increasing TSC protein dosage. Surprisingly, *Adar*^*5G1*^; *ChAT>TSC1*, *TSC2* (Fig. 4a) with reduced signaling to Tor through the insulin pathway do not show strong rescue of *Adar*^*5G1*^mutant locomotion defects. This suggests that any aberrant axonal growth signal in the *Adar*^*5G1*^mutant is not due to alteration in an upstream signal through the insulin receptor, nor through the anaplastic lymphoma kinase that may substitute for insulin receptor in the brain that also signals through PI3K [43] to the Tor complex 1 (TORC1). If suppression of the *Adar* mutant phenotype by reduced Tor is not due to changed responsiveness to external signals such as insulin, then it may be due to an intracellular effect. Since Tor is activated on lysosomes, there may be an aberrant intracellular feedback from autophagy that leads to increased Tor.

To determine whether increased autophagy may be rescuing *Adar* mutant defects by clearing aberrant accumulations of synaptic vesicles, we measured levels of the presynaptic protein Synaptotagmin1 that is associated with the synaptic vesicles in heads of *Adar*^*5G1*^ mutant and rescued flies by immunoblotting. Immunoblotting of head protein extracts with anti-Synaptotagmin 1 antibodies demonstrates that there is an aberrant accumulation of Synaptotagmin 1 in *Adar*^*5G1*^mutant heads [25] (Fig. 4d) that is lowered by reduced *Tor* or by increased *Atg5* expression.

### Increased autophagic vesicles but incomplete clearance of ref(2)p in the *Adar*^*5G1*^ mutant

To assess canonical autophagy in the *Adar*^*5G1*^ mutant and rescues, we examined levels of ref(2)p protein. ref(2)p is the *Drosophila* orthologue of the mammalian p62 canonical autophagy adapter protein (also called Sequestosome1) that brings ubiquitinated cargo to canonical autophagosomes; p62 is degraded in the process and p62 accumulates when canonical autophagy is defective [44]. If canonical autophagy is operating normally in *Adar*^*5G1*^ and increased in heads of *Adar*^*5G1*^*; Tor*^*k17004*^ /+ double mutant or *Adar*^*5G1*^*; ChAT>Atg5* flies, then levels of p62 protein should be normal in *Adar*^*5G1*^ and reduced in the double mutants [45]. However, p62 protein levels are twofold higher than normal in *Adar*^*5G1*^ head protein extracts and increase further in the double mutants (Fig. 4e), in particular with increased Atg5. This suggests that canonical autophagy might not be functioning perfectly in the *Adar*^*5G1*^ mutant background, even though it partially clears excess synaptic vesicle proteins (see below).

**Fig. 5:**
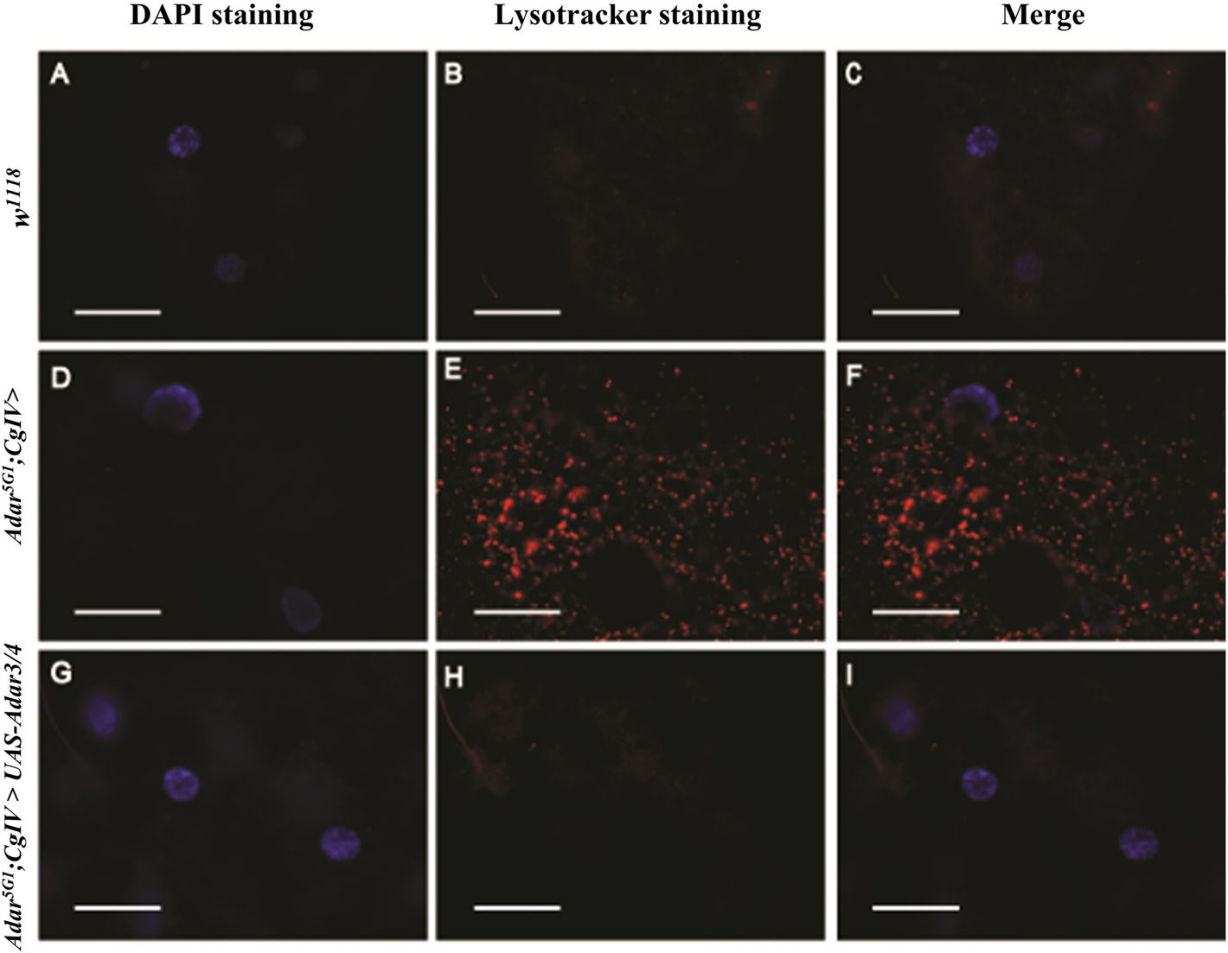
ADAR protein expression rescues the autophagy-related phenotype in *Adar*^*5G1*^ larval fat cells. The fat bodies of **a**–**c** wild-type strain w^1118^, **d**–**f***Adar*^*5G1*^*;CgIV*>, and **g**–**i***Adar*^*5G1*^*;CgIV*>*UAS-Adar3/4* have been dissected and live-stained with DAPI (**a**, **d**, **g**) and Lysotracker (**b**, **e**, **h**) dyes (merges in **c**, **f**, **i**). Wild-type fat body does not show any Lysotracker staining (**b**, **c**). *Adar*^*5G1*^ mutant fat cells have an increased activation of autophagy as indicated by increased Lysotracker staining in lysosomes (**e**, **f**). Expression of the *UAS-Adar3/4* transgene in the *Adar*^*5G1*^ mutant fat cells is sufficient to rescue the elevated basal autophagy (**h**, **i**). Scale bars, 50 μm

Larval fat cells are used to study autophagy in *Drosophila*, as these cells are much larger than brain neurons and form a single sheet of cells in which autophagy is readily induced by starvation of the larvae and detected by staining the lysosomes in live cells with acidic Lysotracker dye. Staining larval fat cells from well-fed larvae of the *Adar*^*5G1*^ mutant with Lysotracker dye shows the presence of increased numbers of lysosomes in the *Adar*^*5G1*^ mutant, even in the absence of starvation (Fig. 5e, f) relative to equivalent wild-type *w*^*1118*^ cells (Fig. 5b, c). Starvation increases the number of lysosomes further in the *Adar*^*5G1*^ mutant cells (data not shown). Expression of *Adar 3/4* (Fig. 5h, i) in *Adar*^*5G1*^ mutant fat cells under the control of the *CollagenIV-GAL4* (*CgIV-GAL4*) driver is sufficient to eliminate the elevated basal autophagy in the *Adar*^*5G1*^ mutant, as indicated by the loss of Lysotracker vesicle staining.

### Rescue of *Adar* mutant phenotypes by increased expression of the endosomal microautophagy (eMI) protein Hsc70-4

**Fig. 6:**
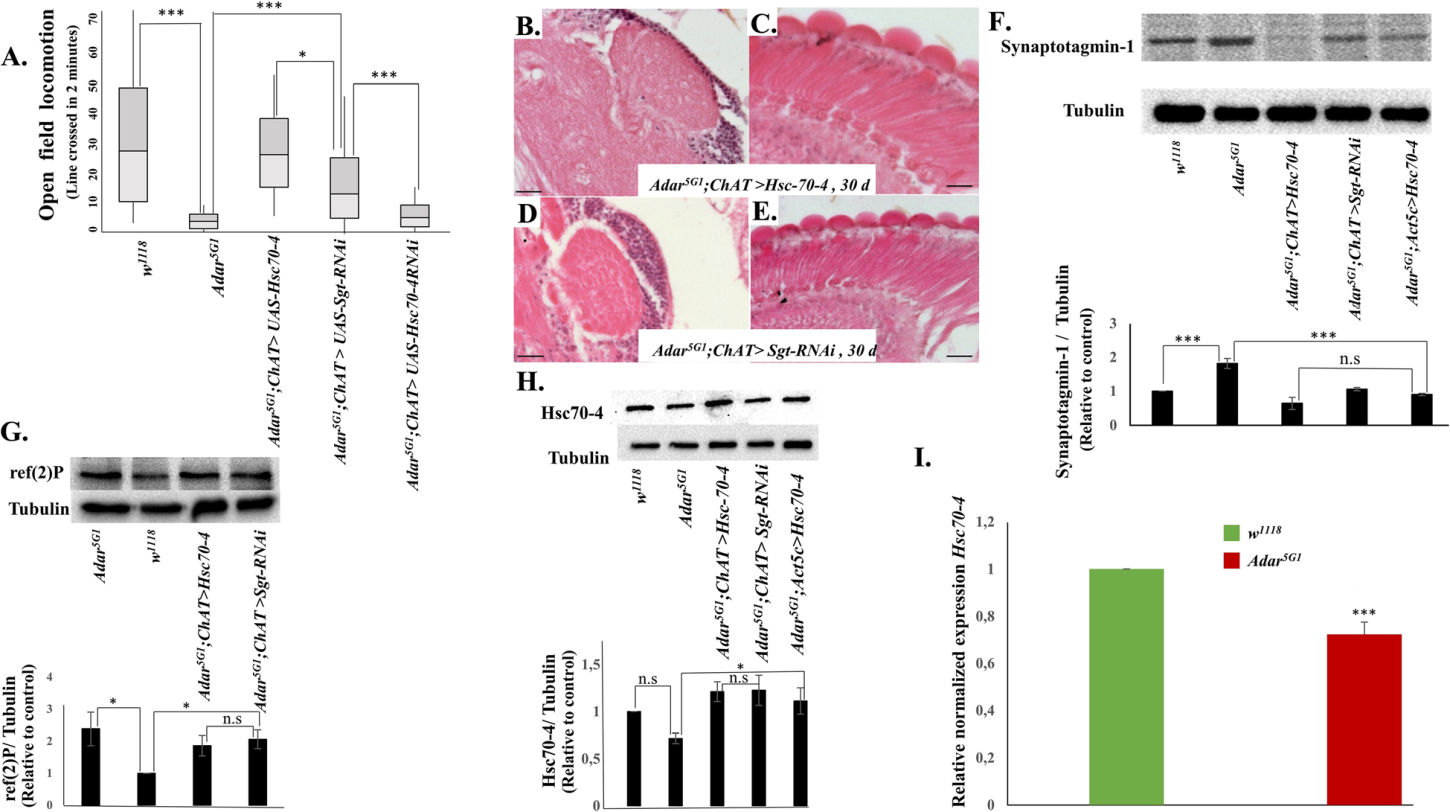
Increased Hsc70-4 suppresses *Adar*^*5G1*^ mutant phenotypes. **a** Rescue of *Adar*^*5G1*^ mutant open field locomotion defects in *Adar*^*5G1*^*; ChAT > Hsc70-4* and *Adar*^*5G1*^*; ChAT > Sgt RNAi* flies with increased endosomal microautophagy. *n* ≥ 10. **b** Representative images of MB calyx (× 40) and **c** retina in 30-day *Adar*^*5G1*^*; ChAT>Hsc70-4* (× 40)*.***d** Representative images of MB calyx (× 40) and **e** retina in 30-day *Adar*^*5G1*^*; ChAT>SgtRNAi* (× 40). **f** Immunoblot detection of the presynaptic protein Synaptotagmin1 in *w*^*1118*^, *Adar*^*5G1*^ mutant, *Adar*^*5G1*^*; ChAT>Hsc70-4*, *Adar*^*5G1*^*; ChAT>Sgt RNAi*, and *Adar*^*5G1*^*; Act5c>Hsc70-4* head protein extracts. Quantitation of the immunoblot data is shown below; levels of Synaptotagmin 1 compared to tubulin in each of the different head protein extracts. *n* ≤ 3. **g** Immunoblot to detect ref(2)p, the *Drosophila* p62 autophagy protein, in total head proteins of *Adar*^*5G1*^ mutant, *w*^*1118*^ wild type, *Adar*^*5G1*^*; ChAT>Hsc70-4*, and *Adar*^*5G1*^*; ChAT>Sgt RNAi* flies. *n* ≤ 3. **h** Immunoblot to detect Hsc70-4 protein in total head protein extracts of *w*^*1118*^ wild type, *Adar*^*5G1*^ mutant, *Adar*^*5G1*^*; ChAT>Hsc70-4*, and *Adar*^*5G1*^*; ChAT>Sgt RNAi* flies and *Adar*^*5G1*^*; Act5c>Hsc70-4*. *n* = 3. **i** qPCR of *Hsc70-4* from *w*^*1118*^ wild type and *Adar*^*5G1*^ heads showing that *Hsc70-4* is significantly decreased in *Adar*^*5G1*^ heads. *n* = 6. *p* values in **a**, **e**, **g,** and **h** were calculated by a one-way ANOVA followed by Tukey’s test. The significance of differences between variables was described based on *p* values: *p value < 0.05, **p value < 0.005, ***p value < 0.001, and n.s (not significant). Error bars: SEM (standard error of mean for biological replicates). *p* values in **h** were calculated by Student’s *t* test. Source data values are included in Additional file 6

Recent studies have shown that a different type of starvation-inducible, Tor-inhibited autophagy called endosomal microautophagy (eMI) occurs in *Drosophila* neurons and is especially important in presynaptic active zones [46–49]. To test whether increased eMI rescues *Adar*^*5G1*^ mutant phenotypes, we used the *ChAT-GAL4* and *Act 5C-GAL4* drivers to increase expression of the Hsc70-4 protein by directing expression of *UAS-Hsc70-4*. Increasing Hsc70-4 in cholinergic neurons increases locomotion (Fig. 6a); on the other hand, knocking down of *Hsc70-4* in cholinergic neurons does not improve the *Adar*^*5G1*^ mutant phenotype (Fig. 6a). When acting as a chaperone for neurotransmitter synaptic vesicles, Hsc70-4 acts together with an interacting partner protein called small glutamine-rich tetratricopeptide repeat protein (Sgt), as an ATP-driven molecular chaperone protein. In eMI, Hsp70-4 acts without Sgt to recruit KFERQ-motif proteins to endosomes [46]. The Sgt protein favors the more general chaperone role of Hsc70-4 in synaptic vesicle cycling and suppresses its function in eMI. Therefore, we also increased eMI with an *UAS-Sgt RNAi* construct to decrease expression of Sgt specifically in cholinergic neurons and this also dramatically suppressed the *Adar*^*5G1*^ mutant locomotion defect (Fig. 5a); knockdown of Sgt with the ubiquitous *Act 5C-GAL4* driver is lethal. Increased eMI in the *Adar*^*5G1*^ mutant background also suppresses neurodegeneration. Overexpression of Hsc70-4 (Fig. 6b, c) or knocking down *Sgt* (Fig. 6d, e) in *Adar*^*5G1*^ with *ChAT-GAL4* suppresses the *Adar*^*5G1*^ mutant neurodegeneration in retina and mushroom body.

Immunoblotting of head protein extracts with anti-Synaptotagmin 1 antibodies demonstrates that the aberrant accumulation of Synaptotagmin 1 in *Adar*^*5G1*^mutant heads (Fig. 6f) is dramatically reduced by increased Hsc70-4 expression. We conclude that increased eMI suppresses the *Adar*^*5G1*^ mutant phenotypes. The reduction of Synaptotagmin 1 to below wild-type levels is surprising, but synaptic vesicle-associated proteins are normally present at levels that probably reflect retention of a reserve of older protein molecules in association with the no-longer readily releasable reserve pool of synaptic vesicles [50–52]. We also see a less dramatic decrease in the level of Synaptotagmin 1 when reducing the level of Tor or overexpressing Atg5 in the *Adar*^*5G1*^ mutant background (Fig. 4d). Increased Atg5 is likely to be lowering Synaptotagmin 1 through increased canonical autophagy and is unlikely to be acting within the eMI pathway as Atg5 has been reported to not be required for eMI [47].

We also examined the level of ref(2)p when overexpressing Hsc70-4 or knocking down Sgt in *Adar*^*5G1*^ (Fig. 6g). We did not observe any significant difference in ref(2)p levels between head extracts of *Adar*^*5G1*^ mutant, *Adar*^*5G1*^*; ChAT > Hsc70-4* or *Adar*^*5G1*^*; ChAT > Sgt RNAi* flies. This suggests that, as expected, increased Hsc70-4 does not increase canonical autophagy or significantly change levels of ref(2)p.

Since increasing eMI suppresses the *Adar*^*5G1*^ mutant phenotypes, it is possible that eMI might be insufficient in *Adar*^*5G1*^. To investigate this, we determined the level of Hsc70-4 protein by immunoblotting head protein extracts (Fig. 6h) and by measuring its expression by qPCR (Fig. 6i). By both methods, we observe a small but significant decrease in Hsc70-4 level in *Adar*^*5G1*^.

## Discussion

RNA editing by Adar is required to maintain the integrity of the CNS in adult *Drosophila* [6]. To find suppressors of the *Adar*^*5G1*^ null mutant phenotype, we performed an initial screen for genetic suppressors that increase the viability of *Adar*^*5G1*^ and discovered a key role for Tor-regulated autophagy in all *Adar* mutant phenotypes (Fig. 1a–c, Fig. 2e–h). Tor protein is abnormally increased in *Adar*^*5G1*^ mutant heads (Fig. 1d); therefore, suppression of *Adar* mutant defects by reduced *Tor* gene dosage is, at least in part, a true rescue, i.e., reducing Tor directly corrects a defect in the *Adar* mutant rather than simply activating some entirely unrelated bypass pathway.

Consistent with an autophagy defect, the *Adar*^*5G1*^ mutant neurodegeneration shows resemblances to neurodegenerations in *Drosophila* models of lysosomal storage diseases, a class of neurodegenerations in which lysosomes accumulate different intracellular components [53]. The most distinctive abnormal intracellular components in the *Adar*^*5G1*^ mutant eyes and brains (Fig. 2a–f), apart from double membrane autophagosomes (Fig. 3f), are the multilamellar membrane whorls (Fig. 3h). These have been identified in cell bodies in other *Drosophila* mutants such as *eggroll* [54], *swiss cheese* [55–57], and *benchwarmer/spinster* [58] and are characteristic of the human neurodegenerative Tay-Sachs disease [53, 59]. The formation of large vacuoles in *Adar* mutant mushroom body calyces might be directly related to accumulation of large numbers of neurotransmitter-containing presynaptic vesicles and associated presynaptic proteins such as Synaptotagmin 1 in the brain [25], which is prevented by reduced *Tor* gene dosage or by increased *Atg5* (Fig. 4d) or increased *Hsc70-4* (Fig. 6e) expression to increase autophagy.

Which type of Tor-regulated autophagy is involved in the suppression of *Adar* mutant phenotypes? Canonical autophagy (CA) is still sufficiently functional to mediate rescue of *Adar*^*5G1*^ mutant phenotypes (Fig. 4a–d), even though it may also be somewhat impaired in *Adar*^*5G1*^. Immunoblots show that ref(2)p protein, the *Drosophila* homolog of the vertebrate p62 adapter for canonical autophagy of ubiquitinated proteins, is increased in *Adar*^*5G1*^ and increased much more with reduced Tor or increased Atg5 (Fig. 4e). *Adar*^*5G1*^ larval fat cells also show increased Lysotracker-positive acidic autophagosomal and lysosomal vesicles (Fig. 5e, f). This impeded CA in *Adar*^*5G1*^ might arise because some proteins that have edited isoforms play important roles in CA [60]. Transcripts of *cacophony* (*cac*) and *straightjacket* (*stj*) encode subunits of the pre-synaptic voltage-gated calcium channel that is also required for fusion of lysosomes with autophagosomes and endosomes. Loss of function mutations of *cac* or *stj* impairs neurotransmission and lysosome function in neurons, leading to some accumulation of p62 protein [61], although it is not known whether loss of only the edited isoforms of these proteins is sufficient to cause any similar defect. Other edited transcripts encoding CA-associated proteins include *Atg14*, *Atg17*, *AMPKalpha*, and *Foxo* (Additional file 4: Table S1); all of these, in addition to probable involvement of edited synaptic vesicle-associated proteins in membrane fusion events in CA [61], suggest that both CA and synaptic vesicle are among processes affected by proteins encoded by edited transcripts in CNS. An additional possible explanation for why ref(2)p clearance is impeded in *Adar*^*5G1*^ is that CA is affected by Dicer-2-mediated aberrant innate antiviral immune induction that occurs in *Adar*^*5G1*^-mutant heads (Deng et al., 2020, Nat. Comms, in press), which is likely to result from accumulated unedited intracellular dsRNA in *Adar*^*5G1*^, paralleling the mouse *Adar1* mutant interferon induction through antiviral dsRNA sensors [62–64]. In mammalian cells, innate immune induction impedes CA by diverting p62 from its role as the receptor for ubiquitinated proteins in CA to instead form a cytoplasmic innate immune signaling platform; p62 and other CA substrates then accumulate because they are less efficiently turned over by CA [60]. This cross-regulation of p62 by innate immune signaling helps to redirect CA to innate immune defense, and it is likely that a similar effect also acts on ref(2)p in *Drosophila*; this could in part account for the *Adar*^*5G1*^ mutant ref(2)p protein accumulation.

The increased ref(2)p in the *Adar* mutant may also lead to the increased Tor activation. In vertebrates, the p62 protein associates with TORC1 on the cytosolic surface of the lysosome; increased p62 contributes to increased Tor activation by intracellular amino acids returning from the lysosome [65]. Aberrant Tor activation through this cell-autonomous pathway in *Drosophila* [66] might explain why we could not mimic the *Tor/+* rescue of *Adar* mutant phenotypes by genetic manipulations that interfere with extracellular hormone and growth-related signaling to TORC1, e.g., by increased expression of the TSC1 and TSC2 proteins that repress Tor in the growth signaling pathways (Fig. 4a).

Endosomal microautophagy (eMI) has recently been described as an important new autophagy pathway involved in proteostasis at presynaptic active zones in *Drosophila* [46, 47]. *Drosophila* eMI targets proteins containing KFERQ motifs to endosomes using the KFERQ-recognition protein (Hsc70-4 in *Drosophila*, HSPA8 in humans) that is also used in lysosomal chaperone-mediated autophagy (CMA) in vertebrates. *Drosophila* is believed to lack CMA as it does not have a homolog of the alternatively spliced isoform of lysosomal LAMP2A protein required to recruit HSPA8 to lysosomes [46, 47]. Increased expression of the key Hsc70-4 protein or decreased Sgt increases eMI and rescues *Adar* mutant locomotion defects (Fig. 6a), neurodegeneration (Fig. 6b–d), and elevated Synaptotagmin 1 levels in *Adar* mutant heads (Fig. 6f), without affecting ref(2)p levels (Fig. 6g). Immunoblots for Hsc70-4 indicate that this protein is at a lower level in *Adar* mutant heads (Fig. 6h, i); this suggests that eMI may be insufficient or suppressed by increased Tor in the *Adar* mutant. Similar to the p62 adapter during CA, the Hsc70-4 cargo selector is believed to also be turned over as KFERQ target proteins are recruited and destroyed during eMI. It is not known how activated Tor suppresses eMI; it has been proposed that Atg1 is also involved [47]; possibly, the reduced Hsc70-4 observed in *Adar*^*5G1*^ is part of the mechanism of eMI suppression by increased Tor.

Since rescue of *Adar* mutant locomotion defects by expression of Adar requires expression of the catalytically active Adar protein, we expected that RNA editing of some target transcript might be essential to rescue locomotion [1]. For instance, editing of the transcript encoding *Synaptotagmin 1* might be required because this leads to production of an edited isoform with a different residue close to those that determine the calcium responsiveness of synaptic vesicle exocytosis, potentially affecting the calcium dependence of the synaptic vesicle cycle [24]. Or editing of the transcript encoding *Synapsin* might be required because this changes an important residue that is phosphorylated by cAMP-dependent protein kinase A (PKA); edited synapsin may limit aberrant synaptic vesicle accumulation and clustering [20, 25]. Therefore, rescue of locomotion defects by reduced Tor or increased autophagy without restoring editing of any target transcript is surprising.

## Conclusion

**Fig. 7:**
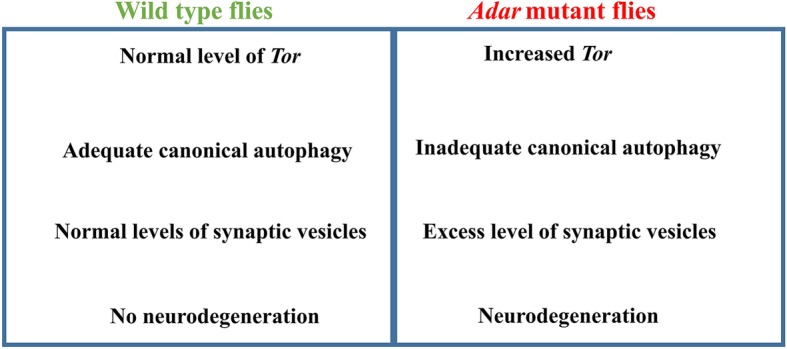
Summary of *Adar* mutant phenotypes. In the *Adar* mutant, aberrantly increased Tor leads to inadequate autophagy, reduced synaptic vesicle clearance, and neurodegeneration

Altering flows of membranes and proteins through Tor-regulated autophagy processes is surprisingly sufficient to overcome *Drosophila Adar* mutant synaptic synaptic defects, locomotion defects, and age-dependent neurodegeneration, presumably by rejuvenating synaptic vesicle pools (these *Adar* mutant defects are summarized in Fig. 7). This suggests that controlling such flows is also a major biological role of Adar RNA editing in *Drosophila*. Can we therefore propose an overall coherent role of ADAR2-type RNA editing in CNS of vertebrates and invertebrates? The independent evolutionary expansions of ADAR2-type RNA editing events in transcripts encoding CNS proteins in advanced insect groups and in cephalopods suggests involvement in brain function and more complex cognition, behavior, and life cycles. In vertebrates, the homologous ADAR2 is a cycling protein that mediates circadian effects [67]; ADAR2 editing also mediates a type of homeostatic postsynaptic plasticity through regulated editing of transcripts encoding glutamate receptor subunits [68, 69], and the seizures that develop in *Adar2* mutant mouse pups also involve widespread effects of aberrant synaptic plasticity [70]. It is likely that *Drosophila* Adar is also involved in circadian rhythms [71], and *Drosophila* Adar is also involved in synaptic plasticity during sleep [25]. Aberrantly increased sleep drive arises because the increased reserve pools of presynaptic neurotransmitter synaptic vesicles cannot be reduced normally during sleep. The role of Adar we outline here acts to protect the brain through effects on synaptic plasticity. Adar RNA editing may be involved in circadian changes in synaptic plasticity and may even mediate beneficial effects of sleep on the brain.

## Methods

### Fly maintenance and fly strains

All fly stocks were raised on standard corn meal-agar medium. Fly stocks were maintained at 18 °C, and crosses were performed at 25 °C. Flies used in aging experiments were maintained in tubes not supplemented with additional yeast, to prevent flies from becoming stuck to the yeast. A single fly was maintained in a vial, and each vial was tipped-on daily. The wild-type control strains were either *w*^*1118*^. The GAL4 driver lines and balancer lines were obtained from the Bloomington Stock Centre. Detailed genotypes of individual strains used are as follows;

- *Tor*^*k17004*^*: y[1] w[67c23]; P{w[+mC]=lacW}Tor[k17004]/CyO,*
  *Tor*^*MB07988*^*: w[1118]; Mi*{ET1}*Tor[MB07988]*
  *S6K*^*KQ*^ (dominant negative): *w[1118]; P{w[+mC]=UAS-S6k.KQ}2*
  Thor: w[*]; P{w[+mC]=UAS-Thor.wt}2
  *Atg6: y; UAS-Atg6-2D; Sb/Tm6b* (from U. Pandey)
  *Atg5: y[1] w[1118]; wg[Sp-1]/CyO; P{w[+mC]=UAS-eGFP-drAtg5}16*
  *Atg1[6A]: y,w,hsflp;; UAS-Atg1[6A],* (from T. Neufeld)
  *Atg1[GS10797](EP line): y,w,hsflp; Atg1[GS10797],* (from T. Neufeld)
- *TSC1, TSC2: y,w,hsFlp; UAS-TSC1, UAS-TSC2,* (from T. Neufeld)
- *UAS-Hsc70-4: w[126]; P{w[+mC]=UAS-Hsc70-4.K71S}G*
- *BDSC #28709 -**y*^*1*^*v*^*1*^*;**P{TRiP.JF03136}attP2**( Hsc70-4 RNAi)*
- *BDSC #**61267**-**y*^*1*^*v*^*1*^*;**P{TRiP.HMJ23046}attP40**(sgt RNAi)*

The GAL4 binary system was used to express transgenes in the *Adar* mutant background. The *Adar*^*5G1*^ mutant strain was combined with *ChAT>-GAL4*, and virgin females of these strains were crossed to males of the transgenic lines bearing the *Drosophila UAS-cDNA* constructs. Female genotype is *y, Adar*^*5G1*^*, w / w, FM7, Bar; (ChAT-GAL4.7.4)19B,(UASGFP.S65T)T2 / (ChATGAL4.7.4)19B,(UASGFP.S65T)T2*.

### *DrosDel* screen for suppressors of reduced viability in the *Adar*^*5G1*^ mutant

To screen for suppressors of *Adar*^*5G1*^ mutant reduced viability, we crossed virgin female *y, Adar*^*5G1*^*, w /FM7, Bar* in groups of five with males from the *DrosDel / SM5 Cy* lines. Taking male non-*Curly* progeny, we counted the *Adar*^*5G1*^*; DrosDel* / + and *FM7 Bar; DrosDel / +* flies that eclosed from pupae and determined the ratio of male *y, Adar*^*5G1*^*, w; Df / +* to sibling male *FM7; Df* / +progeny for each deficiency. *DrosDel* deficiencies are marked with mini-*w*^*+*^*.* Tests of *Tor* mutants were performed in the same way.

### Open field locomotion assays

Open field locomotion was measured by recording crossing of individual flies over lines in a gridded Petri dish (three 2-min measurements on each of 10 or more flies for each line) as previously described [17]. The data are presented as the average number of lines crossed by a fly in the 2-min period. The flies are collected on the day of eclosion from the pupae. Next morning, when effects of CO_2_ anesthesia have worn off, they are individually introduced to the measuring dish and the measuring period begins after tapping the dish once on the bench. The test measures the flies maximized movement response to an initial stimulation and to a new environment.

### Histology techniques

For standard hematoxylin-eosin stained sections, *Drosophila* heads were fixed at room temperature in Carnoy’s fixative for 4 h. For detecting cell death, the terminal deoxynucleotidyl transferase Biotin-dUTP nick end-labelling (TUNEL) kit from Roche was used. *Drosophila* heads were fixed for 4 h at room temperature in 4% paraformaldehyde. The heads were embedded into paraffin wax with standard histology procedures. Sections were cut at 6 μm and either stained with hematoxylin and eosin for pathological analysis or stained for cell death according to the TUNEL kit instructions. Images were captured using a compound microscope, which comprised a Coolsnap HQ CCD camera (Photometrics Ltd., Tucson, AZ) with Plan-neofluar objectives (Carl Zeiss, Welwyn Garden City, UK). Images were captured with neofluar objectives at × 40 (with a numerical aperture of 1.3) for eyes and at × 63 and × 40 (with a numerical aperture of 1.25) for mushroom bodies. Color additive filters (Andover Corporation, Salem, NH) installed in a motorized filter wheel (Ludl Electronic Products, Hawthorne, NY) were used sequentially to collect red, green, and blue images, which were then superimposed to form a color image. Image capture and analysis were performed with in-house scripts written for IPLab Spectrum (Scanalytics Corp, Fairfax, VA). The brightness and contrast were altered with the advanced histogram section in either IP Lab Spectrum or Adobe Photoshop. This was done by manually setting the minimum and maximum pixel intensities on the histogram. If necessary, the gamma was altered on the histogram. The images shown are representative examples from samples of 10–20 heads sectioned for each age and genotype.

### Electron microscopy

The *Adar*^*5G1*^ mutants and *w*^*1118*^ controls were aged to 25 days or longer from parallel collections. The proboscis was removed in Schneider’s insect media, and the heads were fixed for at least 1 h in 2.5% glutaraldehyde and subsequently fixed in 1% osmium tetroxide in Sorenson’s buffer. The heads were dehydrated and embedded into resin. Survey sections of 0.5 μm were cut through the frontal brain, and ultra-thin sections were cut at the regions of interest. The sections were stained with 2% aqueous uranyl acetate for 15 min and lead citrate (supplied by Leica) for 5 min. The tissue sections were viewed with a Philips CM 100 Compustage (FEI) transmission electron microscope, and digital images are collected with an AMT CCD camera (Deben). The brightness and contrast were altered manually with the advanced histogram section in either IP Lab Spectrum or Adobe Photoshop by setting the minimum and maximum pixel intensities on the histogram. If necessary, the gamma was altered on the histogram.

### Immunoblotting

Male flies (minimum 15 flies) of the desired genotype were collected and aged for 2 days and then homogenized in NB Buffer (150 mM NaCl, 50 mM Tris-HCl pH 7.5, 2 mM EDTA, 0.1% NP-40). Protein concentration was determined with Pierce BCA Protein Assay Kit. An equal amount of protein was loaded in each lane of a Tris-Glycine Gel and transferred to a nitrocellulose membrane. The membrane was blocked with 5% BSA, incubated with primary antibody overnight. The next day, the membrane was incubated with secondary antibody and developed with Pierce ECL Western Blotting Substrate. Anti-Ref2P (antibody registry ID: AB_2570151 (1:1000) was a gift from Tor Erik Rusten (University of Oslo), anti-synaptotagmin (1:500) (Developmental Studies Hybridoma Bank, DSHB Hybridoma Product 3H2 2D7, Antibody Registry ID: AB_528483), anti-Hsc70-4 (1;1000) was a gift from Konrad Zinsmaier (Bronk et.al, Neuron 2001), anti-Tor antibody (antibody registry ID: AB_2568971) (1:1000) was a gift from Gábor Juhász, anti-Tublin (Developmental Studies Hybridoma Bank, DSHB Hybridoma Product 12G10, antibody registry ID: AB_1157911) (1:5000). Imaging was performed with ChemiDoc™ XRS+ System, signal intensity was quantified with Image J software, and statistical analyses were done with the *t* test.

### qPCR

RNA from approximately 20 fly heads was isolated with Tripure, and cDNA generated with RevertAid First Strand cDNA Synthesis Kit (Thermo Scientific). qPCR reactions were performed with The LightCycler® 480 SYBR Green I Master mix, and the primers listed in Additional file 5: Table S2 were used to measure expression levels. Expression levels were normalized to those of RP49, and *t* tests were used for statistical analysis.

### Lysotracker staining of larval fat cells

*Drosophila* larvae were collected, and brains and fat body dissected in cold PBS. The tissue of interest was incubated with LysoTracker® Red DND-99, Molecular Probes, Invitrogen (l μl of dye in 10 ml of cold PBS), for 5 min in ice. After five 2-min washes in PBS, the tissue was mounted in Vectashield DAPI and viewed with a fluorescent microscope.

### Statistical analyses

Two sample data were analyzed by Student’s *t* test. A *p* value of < 0.05 was considered statistically significant. In more than three groups, *p* values were calculated by a one-way ANOVA followed by Tukey’s test. The significance of differences between variables was described based on *p* values: *p value < 0.05, **p value < 0.005, ***p value < 0.001, and n.s (not significant). Error bars: SEM (standard error of mean for biological replicates).

## Supplementary information

**Additional file 1: Figure S1.** Screen of *DrosDel* deletions on Chromosome 2 L for rescue of *Adar*^*5G1*^ viability. Ratio of *Adar*^*5G1*^ to *FM7 Bar* genotypes among male progeny in the presence of *DrosDel* deficiencies, or in their absence (*w*^*1118*^ cross at the bottom) (expressed as a percentage). Progeny are obtained by crossing *Adar*^*5G1*^*/ FM7, Bar* virgin females to males to *w*^*1118*^ males or males of *DrosDel/SM5, Cy* deficiency stocks.

**Additional file 2: Figure S2.***Adar*^*5G1*^ neurodegeneration at 30 days. Images of 6 micron thick haematoxylin and eosin stained sections through mushroom body calyces (left panels, (63X)) and retinas (right panels, 40X) of 30-day *Adar*^*5G1*^.

**Additional file 3: Figure S3.** Neuronal cell death is not prominent in heads of 25-day-old *Adar*^*5G1*^ mutant flies. (A) TUNEL staining to detect apoptotic cells in head sections from 25-day-old *Adar*^*5G1*^ mutant flies stained with DAPI to detect nuclei. TUNEL-positive nuclei are not detected in neurons However TUNEL-positive nuclei are conspicuous in head fat bodies of 25-day-old *Adar*^*5G1*^ mutant flies (boxed area in A). (B) Magnification of area boxed in A (C) Haematoxylin and eosin stained section serial to A, white box indicates fat body tissue. (D) Magnification of area boxed in C. (E, F) Images show representative 6 micron thick haematoxylin and eosin stained sections through mushroom body calyces (left panels, (63X)) and retinas (right panels, 40X) of 30-day *Adar*^*5G1*^; *ChAT>UAS-p35.* Scale bars: 20 μm.

**Additional file 4: Table S1.** List of Adar edited transcripts encoding proteins required for autophagy.

**Additional file 5: Table S2.** Primers used for qPCR.

**Additional file 6.** Excel sheet containing source data file for Figure 1A ,1B ,1C.1D ,4A,4D,4E,6A,6F,6G,6H and 6I.

## Data Availability

All data generated or analyzed during this study are included in this published article and its supplementary information files.
