# Supplementary material for: Membrane and synaptic defects leading to neurodegeneration in Adar mutant Drosophila are rescued by increased autophagy
Source: BMC Biol. 2020 Feb 14;18:15. doi: 10.1186/s12915-020-0747-0 (PMC7020516; doi:10.1186/s12915-020-0747-0)
Supplement: Supplementary file 3 — Additional file 1: Figure S1. Screen of DrosDel deletions on Chromosome 2 L for rescue of Adar5G1 viability. Ratio of Adar5G1 to FM7 Bar genotypes among male progeny in the presence of DrosDel deficiencies, or in their absence (w1118 cross at the bottom) (expressed as a percentage). Progeny are obtained by crossing Adar5G1/ FM7, Bar virgin females to males to w1118 males or males of DrosDel/SM5, Cy deficiency stocks. [file 12915_2020_747_MOESM1_ESM.pdf]

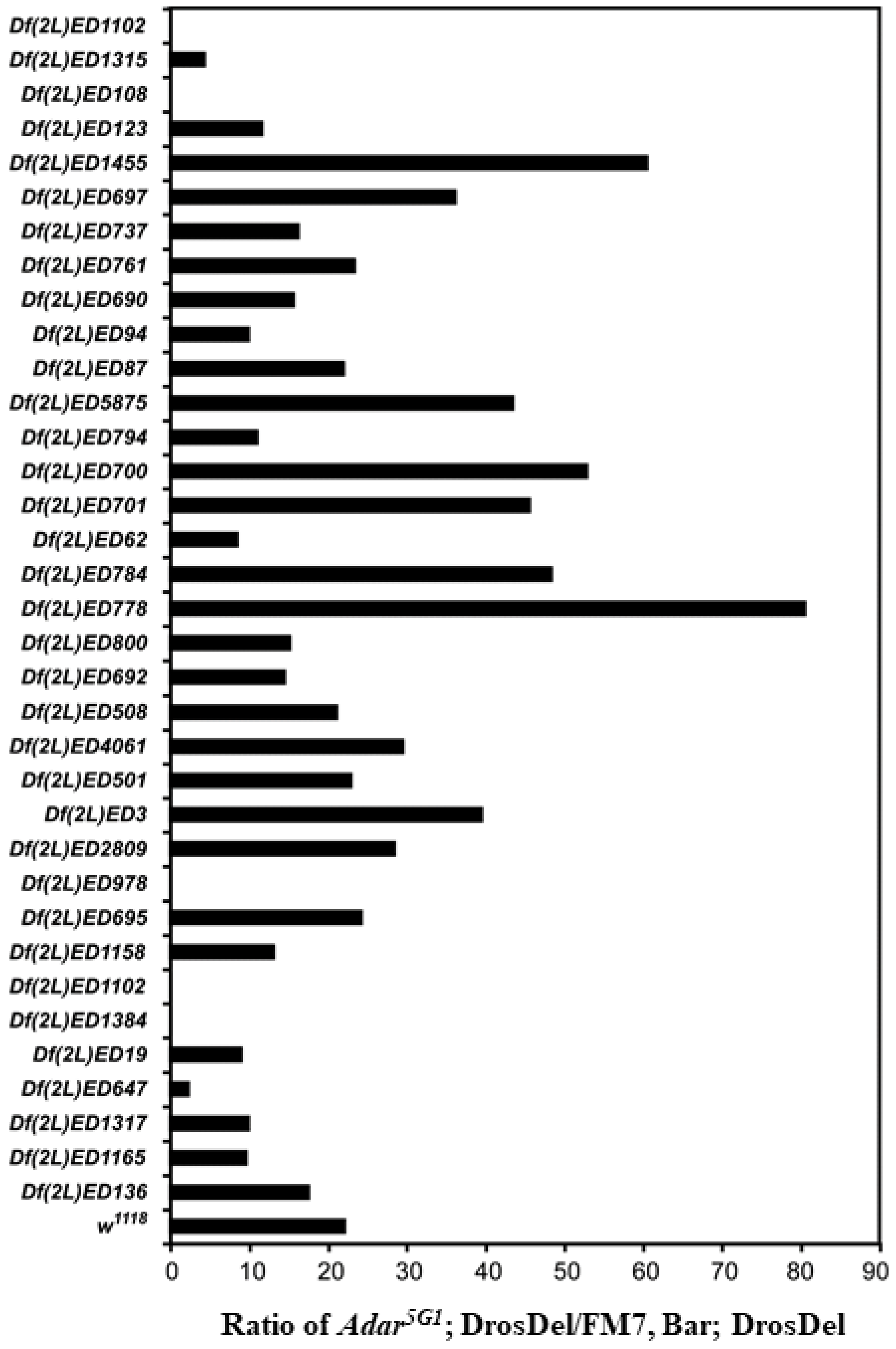

Supplementary Figure S1. Screen of *DrosDel* deletions on Chromosome 2L for rescue of *Adar*^{5G1} viability. Ratio of *Adar*^{5G1} to *FM7 Bar* genotypes among male progeny in the presence of *DrosDel* deficiencies, or in their absence (w^{1118} cross at the bottom) (expressed as a percentage). Progeny are obtained by crossing *Adar*^{5G1} / *FM7, Bar* virgin females to males to w^{1118} males or males of *DrosDel/SM5, Cy* deficiency stocks.
