# Supplementary material for: Membrane and synaptic defects leading to neurodegeneration in Adar mutant Drosophila are rescued by increased autophagy
Source: BMC Biol. 2020 Feb 14;18:15. doi: 10.1186/s12915-020-0747-0 (PMC7020516; doi:10.1186/s12915-020-0747-0)
Supplement: Supplementary file 4 — Additional file 2: Figure S2.Adar5G1 neurodegeneration at 30 days. Images of 6 micron thick haematoxylin and eosin stained sections through mushroom body calyces (left panels, (63X)) and retinas (right panels, 40X) of 30-day Adar5G1. [file 12915_2020_747_MOESM2_ESM.pdf]

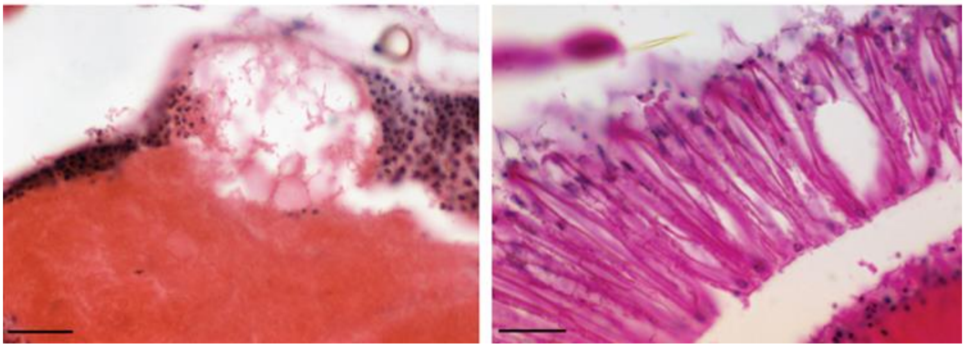

Supplementary Figure S2. ***Adar*^{5G1} neurodegeneration at 30 days.**

Images of 6 micron thick haematoxylin and eosin stained sections through mushroom body calyces (left panels, (63X)) and retinas (right panels, 40X) of 30-day *Adar*^{5G1}.
