# Supplementary material for: Membrane and synaptic defects leading to neurodegeneration in Adar mutant Drosophila are rescued by increased autophagy
Source: BMC Biol. 2020 Feb 14;18:15. doi: 10.1186/s12915-020-0747-0 (PMC7020516; doi:10.1186/s12915-020-0747-0)
Supplement: Supplementary file 5 — Additional file 3: Figure S3. Neuronal cell death is not prominent in heads of 25-day-old Adar5G1 mutant flies. (A) TUNEL staining to detect apoptotic cells in head sections from 25-day-old Adar5G1 mutant flies stained with DAPI to detect nuclei. TUNEL-positive nuclei are not detected in neurons However TUNEL-positive nuclei are conspicuous in head fat bodies of 25-day-old Adar5G1 mutant flies (boxed area in A). (B) Magnification of area boxed in A (C) Haematoxylin and eosin stained section serial to A, white box indicates fat body tissue. (D) Magnification of area boxed in C. (E, F) Images show representative 6 micron thick haematoxylin and eosin stained sections through mushroom body calyces (left panels, (63X)) and retinas (right panels, 40X) of 30-day Adar5G1; ChAT>UAS-p35. Scale bars: 20 μm. [file 12915_2020_747_MOESM3_ESM.pdf]

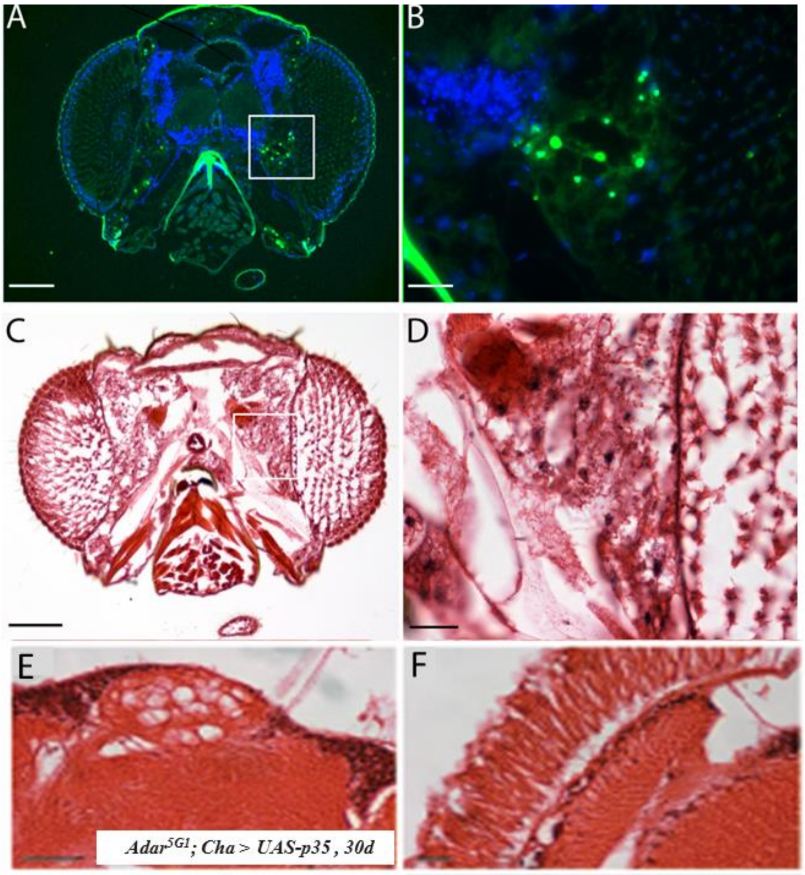

Supplementary Figure S3. Neutronal cell death is not prominent in heads of 25-day-old *Adar*^{5G1} mutant flies. (A) TUNEL staining to detect apoptotic cells in head sections from 25-day-old *Adar*^{5G1} mutant flies stained with DAPI to detect nuclei. TUNEL-positive nuclei are not detected in neurons However TUNEL-positive nuclei are conspicuous in head fat bodies of 25-day-old *Adar*^{5G1} mutant flies (boxed area in A). (B) Magnification of area boxed in A (C) Haematoxylin and eosin stained section serial to A, white box indicates fat body tissue. (D) Magnification of area boxed in C. (E, F) Images show representative 6 micron thick haematoxylin and eosin stained sections through mushroom body calyces (left panels, (63X)) and retinas (right panels, 40X) of 30-day *Adar*^{5G1}; *ChAT>UAS-p35*. Scale bars: 20μ**m**
