# Supplementary material for: Membrane and synaptic defects leading to neurodegeneration in Adar mutant Drosophila are rescued by increased autophagy
Source: BMC Biol. 2020 Feb 14;18:15. doi: 10.1186/s12915-020-0747-0 (PMC7020516; doi:10.1186/s12915-020-0747-0)
Supplement: Supplementary file 6 — Additional file 4: Table S1. List of Adar edited transcripts encoding proteins required for autophagy. [file 12915_2020_747_MOESM4_ESM.pdf]

	NAME	SYMBOL	GO BIOLOGICAL PROCESS (Flybase)
**1**	***SNF4/AMP-activated protein kinase gamma subunit***	***SNF4Agamma***	**autophagy | inferred from mutant phenotype**
**2**	***Gigyf***	***Gyf***	**regulation of autophagy | inferred from genetic interaction with Atg1, Atg13**
**3**	***Nitrogen permease regulator-like 3***	***Nprl3***	**negative regulation of TOR signaling | inferred from mutant phenotype**
**4**	***multiple ankyrin repeats single KH domain***	***mask***	**inferred from mutant phenotype inferred from genetic interaction with Atg18a**
**5**	***AMP-activated protein kinase alpha subunit***	***AMPKalpha***	**negative regulation of TOR signaling |**
**6**	***Protein modification; protein ubiquitination***	***hiw***	**autophagy | inferred from direct assay**
**7**	***Acinus***	***Acn***	**regulation of autophagy | inferred from mutant phenotype**
**8**	***Autophagy-related 17***	***Atg17***	**autophagy | inferred from mutant phenotype inferred from genetic interaction with Atg1**
**9**	***Autophagy-related 14***	***Atg14***	**regulation of autophagy | inferred from mutant phenotype**
**10**	***forkhead box, sub-group O***	***foxo***	**regulation of autophagy | inferred from mutant phenotype**
**11**	***straightjacket***	***stj***	**autophagosome maturation | inferred from mutant phenotype**
**12**	***mauve***	***mv***	**negative regulation of autophagosome size | inferred from mutant phenotype**
**13**	***Darkener of apricot***	***Doa***	**positive regulation of autophagy| inferred from mutant phenotype**
**14**	***similar***	***sima***	**positive regulation of autophagy| inferred from mutant phenotype**
**15**	***Ecdysone receptor***	***EcR***	**regulation of autophagy | inferred from mutant phenotype**
**16**	***Protein phosphatase 2A at 29B***	***Pp2A-29B***	**autophagy | inferred from mutant phenotype**
**17**	***Adaptor Protein complex 2, alpha subunit***	***AP-2alpha***	**positive regulation of autophagy| inferred from mutant phenotype**
**18**	***cacophony***	***cac***	**autophagosome maturation | inferred from mutant phenotype**

**19**	***rolled***	***rl***	**macroautophagy | inferred from mutant phenotype**
**20**	***spaghetti-squash activator***	***sqa***	**autophagy | inferred from mutant phenotype**
**21**	***Ubiquitin-63E***	***Ubi-p63E***	**autophagy | inferred from mutant phenotype**
**22**	***widerborst***	***wdb***	**autophagy | inferred from mutant phenotype**

Supplementary Table 1: List of Adar edited transcripts encoding proteins required for autophagy. Source: FlyBase.
