# Supplementary material for: Membrane and synaptic defects leading to neurodegeneration in Adar mutant Drosophila are rescued by increased autophagy
Source: BMC Biol. 2020 Feb 14;18:15. doi: 10.1186/s12915-020-0747-0 (PMC7020516; doi:10.1186/s12915-020-0747-0)
Supplement: Supplementary file 7 — Additional file 5: Table S2. Primers used for qPCR. [file 12915_2020_747_MOESM5_ESM.pdf]

Hsc70-4 forward Primer	CCAGGGTAATCGTACCACTCC
Hsc70-4 Reverse Primer	GCGTCGAAGATCGTCTGGG

Supplementary Table 2: Primers used for qPCR
